# Atypical cause of a typical chest pain

**DOI:** 10.12669/pjms.303.5440

**Published:** 2014

**Authors:** Imran Haider

**Affiliations:** 1 Imran Haider, MD, Saint Luke’s Hospital, Department of Internal Medicine, Saint Louis, MO, USA.

A 56 year old African American female who had a past medical history of transient ischemic attack in 2006 presented in our emergency department with first episode of sudden onset of severe central chest pain radiating to both arms , exaggerated by exertion, without any relieving factors, associated with nausea and sweating. She was taking baby aspirin, folic acid and multivitamin regularly. She did not have any history of smoking, alcohol or drug abuse.

On Examination, she was in moderate distress due to pain, her pulse rate was 110 per minute (regular), and blood pressure was 153/85 mm HG. She was having mild tenderness in left upper quadrant. Rest of the physical examination was benign.

Her initial electrocardiogram (EKG) did not show any acute ST segment or T wave changes. Troponin-I was also negative. She was started on oxygen, intra venous morphine and normal saline and shifted to the medical floor for further management. Her serial EKG’s and troponin’s remain negative. Transthoracic echocardiogram did not show any evidence of wall motion abnormalities. Further laboratory data showed that she was having hemolytic anemia with hemoglobin of 9.7 gm/dl and reticulocyte count of 4.8%.

Upon further digging into the history, she mentioned that she had been periodically visiting hematology clinic, although she did not remember the reason for that. Once we contacted the hematologist, he revealed that she was diagnosed with Hemoglobin SC (HB-SC) disease with hemoglobin electrophoresis almost 2 years ago. Her blood counts had been stable over the period of time and she never had a painful crisis.

Differential diagnosis at this point was chest syndrome, splenic infarction and acute painful crisis. Her initial chest X-Ray and computed tomography (CT) of the chest did not show any evidence of consolidation, and she was hemodynamically stable, so chest syndrome was ruled out. CT of the abdomen showed mild splenomegaly without any evidence of micro infarction. She responded to the above management quite nicely, so the diagnosis of acute painful crisis due to hemolytic anemia of underlying HBSC disease was established.

Individuals with HB-SC disease are heterozygous with a 50:50 mixture of HB-S and HB-C. They do not typically present with acute painful crisis because HB-C molecule does not polymerize as readily as HB-S, so they have fewer sickle cells and mild degree of hemolysis.^1^ As compared with HB-SS disease, these patients have same life time complications but with a lesser frequency and severity.^2^ They have a higher incidence of osteonecrosis and peripheral retinopathy, so they need monitoring for that.^3^

## Notes

***Source(s) of support:*** None.
